# Factores pronósticos de gravedad de la infección por SARS-CoV-2

**DOI:** 10.1515/almed-2020-0069

**Published:** 2020-11-13

**Authors:** Ricardo Rubio Sánchez, Esperanza Lepe Balsalobre, María del Mar Viloria Peñas

**Affiliations:** UGC Laboratorio Clínico, Hospital Universitario Virgen de Valme, Área de Gestión Sanitaria Sur de Sevilla, Sevilla, España

**Keywords:** coronavirus, COVID-19, gravedad, infección, laboratorio, SARS-CoV-2

## Abstract

**Objetivos:**

El SARS-CoV-2 es un nuevo coronavirus, responsable de la enfermedad COVID-19. Entre las alteraciones de los parámetros de laboratorio se han descrito leucocitosis con linfopenia, neutrofilia y aumento de dímero D, proteína C reactiva, ferritina, procalcitonina y lactato deshidrogenasa. El objetivo de este estudio ha sido describir las características clínicas y los parámetros de laboratorio de pacientes ingresados con infección por SARS-CoV-2 e identificar factores pronósticos de progresión de la enfermedad.

**Materiales:**

Los pacientes incluidos en el estudio fueron clasificados en dos grupos en función de la gravedad de la infección. Los variables estudiadas fueron los datos demográficos, antecedentes personales, días de ingreso hospitalario, evolución del cuadro neumónico, tratamiento farmacológico y parámetros analíticos. Se realizó un análisis descriptivo de los datos recogidos, así como un análisis multivariante para identificar factores pronósticos de gravedad de la infección.

**Resultados:**

La población de este estudio incluyó a 197 pacientes, considerándose 127 leves y 70 graves. Se encontraron diferencias estadísticamente significativas entre los dos grupos en la mayoría de los parámetros de laboratorio. El análisis multivariante incluyó como factores pronósticos de gravedad la edad avanzada, niveles elevados de leucocitos y concentraciones aumentadas de proteína C reactiva (PCR), γ-glutamiltransferasa (GGT) y procalcitonina (PCT) en el momento del ingreso hospitalario.

**Conclusiones:**

Los factores pronósticos de gravedad de la infección por SARS-CoV-2 identificados en este estudio (edad, leucocitos, PCR, GGT y PCT) permiten predecir el curso de la enfermedad en las etapas iniciales.

## Introducción

El SARS-CoV-2 es un nuevo tipo de virus de la familia *Coronaviridae* que fue identificado en enero de 2020 en humanos con clínica de neumonía viral en Wuhan, China. Este virus pertenece al género betacoronavirus, en el que también se incluyen los coronavirus responsables del síndrome respiratorio agudo grave (SARS-CoV) y el síndrome respiratorio de Oriente Medio (MERS-CoV) [1]. El 11 de marzo de 2020, la Organización Mundial de la Salud (OMS) declaró la pandemia mundial [1], [2].

La enfermedad que causa el SARS-CoV-2 ha sido denominada COVID-19 y entre las manifestaciones clínicas más frecuentes se incluyen fiebre, tos y disnea. Sin embargo, esta enfermedad se caracteriza por un espectro clínico variado que abarca desde un estado asintomático hasta un cuadro grave caracterizado por neumonía intersticial y síndrome respiratorio agudo severo en aproximadamente el 20% de los pacientes. Además, la enfermedad puede progresar a insuficiencia respiratoria con disfunción multiorgánica y muerte, especialmente en personas de edad avanzada o con comorbilidades subyacentes. Por lo tanto, es de especial relevancia el estudio de biomarcadores para el diagnóstico, así como la identificación de predictores de gravedad de la infección por SARS-CoV-2 [3], [4].

La infección por SARS-CoV-2 provoca la activación del sistema inmune innato generando una respuesta excesiva y causando la desregulación de la cascada de citoquinas. Lo anterior ocasiona daño en el sistema microvascular, activación del sistema de coagulación e inhibición de la fibrinolisis. Los pacientes con COVID-19 presentan niveles más elevados de citoquinas proinflamatorias asociadas con el agotamiento de las células T, la inflamación pulmonar y el daño pulmonar extenso [5], [6]. Diferentes estudios han comunicado niveles aumentados de reactantes de fase aguda que conducen a una disfunción hepática y un proceso de coagulación intravascular diseminada en pacientes con infección por SARS-CoV-2 [6], [7].

Con respecto a parámetros de laboratorio, la leucocitosis con linfopenia, probablemente causada por la translocación de linfocitos de sangre periférica a pulmones, y la neutrofilia fueron comunes en casos de infección. Además, en pacientes infectados por SARS-CoV-2, se ha observado un aumento de dímero D, velocidad de sedimentación globular, proteína C reactiva, ferritina, procalcitonina, transaminasas, bilirrubina, creatinina y lactato deshidrogenasa. En estos pacientes también se ha descrito una disminución en la concentración de plaquetas y hemoglobina, así como un aumento en el tiempo de protrombina [2], [5], [6], [7], [8].

El objetivo de este estudio ha sido describir las características clínicas y los parámetros de laboratorio de pacientes ingresados con infección por SARS-CoV-2 en la provincia de Sevilla e identificar factores pronósticos de progresión de la enfermedad.

## Materiales y métodos

### Diseño del estudio y pacientes

Estudio descriptivo, retrospectivo y de corte transversal realizado en el Hospital Universitario Virgen de Valme (Sevilla, España) que incluyó a pacientes con infección por SARS-CoV-2 ingresados entre el 14 de marzo y el 5 de junio de 2020. Este trabajo sigue las recomendaciones éticas de la Declaración de Helsinki y fue aprobado por el Comité Ético de Investigación Clínica Sevilla Sur (N.º 1447-N-20).

Los criterios de inclusión empleados fueron los siguientes: pacientes de cualquier edad y sexo con infección por SARS-CoV-2 confirmada por reacción en cadena de la polimerasa de transcripción inversa (RT-PCR) y con ingreso hospitalario. Se excluyeron del estudio aquellos pacientes con sospecha de infección, pero RT-PCR negativa.

Los pacientes incluidos en el estudio fueron clasificados en dos grupos en función de la gravedad de la infección: pacientes leves (ingreso en planta hospitalaria) y pacientes graves (ingreso en Unidad de Cuidados Intensivos (UCI) y/o exitus).

### Datos recogidos

Los datos clínicos, los registros de enfermería y los resultados de laboratorio fueron recogidos de la historia clínica digital de los pacientes. Dos investigadores independientes al estudio revisaron todos los datos recogidos. Los variables estudiadas fueron las siguientes:

– Datos demográficos: edad y sexo.
– Antecedentes personales: hipertensión arterial, dislipemia, enfermedad cardiovascular, diabetes mellitus, insuficiencia renal crónica, cáncer, enfermedad vascular, enfermedad pulmonar, patología tiroidea, enfermedad hepática y anemia.
– Días de ingreso hospitalario, duración de la estancia en UCI y evolución del cuadro neumónico.
– Tratamiento farmacológico: hidroxicloroquina, lopinavir/ritonavir, corticoides, azitromicina, tocilizumab, interferón β, ciclosporina y anakinra.
– Parámetros analíticos: leucocitos, linfocitos, neutrófilos, hemoglobina, plaquetas, velocidad de sedimentación globular (VSG), tiempo de protrombina (TP), tiempo de tromboplastina parcial activada (TTPA), dímero D, creatinina, bilirrubina total, alanina aminotransferasa (ALT), aspartato aminotransferasa (AST), γ-glutamiltransferasa (GGT), lactato deshidrogenasa (LDH), proteína C reactiva (PCR), procalcitonina (PCT) y ferritina.

### Análisis de laboratorio

Se obtuvieron muestras de sangre y una muestra nasofaríngea de cada paciente incluido en el estudio. La muestra de sangre fue recogida por venopunción en tres tubos diferentes: uno con el anticoagulante EDTA para el recuento de células sanguíneas y la VSG, empleando el analizador Sysmex XN-2000 (Sysmex, Kobe, Japón); otro tubo con citrato sódico para el estudio del TP, TTPA y dímero D en el equipo Sysmex CS-5100 (Sysmex, Kobe, Japón) por inmunoensayo; y un último tubo con heparina de litio para el análisis en plasma de parámetros bioquímicos en el analizador modular Hitachi Cobas c702 (Roche Diagnostics, Rotkreuz, Suiza): creatinina, bilirrubina total, ALT, AST, GGT y LDH por fotometría, mientras que PCR, PCT y ferritina por inmunoturbidimetría.

Para el exudado nasofaríngeo se emplearon hisopos de garganta en medio molecular Copan eNATM para RT-PCR para SARS-CoV-2. Los ARN virales se extrajeron de las muestras usando el kit de aislamiento de ARN compacto puro MagNA (Roche Diagnostics, Rotkreuz, Suiza) y se realizó una RT-PCR cuantitativa usando los cebadores y las sondas dirigidas a los genes *ORF1ab* y *N* de SARS-CoV-2, según lo recomendado por los Centros para el Control y Prevención de Enfermedades, utilizando un kit comercial específico para la detección de SARS-CoV-2 (VIASURE CerTest BIOTEC). Se usaron cebadores específicos y una sonda marcada con fluorescencia. Las muestras se consideraron positivas si se detectaba señal de amplificación en el sistema de detección y en el control interno. Las muestras se consideraron negativas si la muestra no mostraba señal de amplificación en el sistema de detección, pero el control interno era positivo. Se repitieron las muestras con una señal de amplificación de control negativo o ausencia de señal en el positivo.

### Análisis estadístico

Los datos fueron procesados con el programa SPSS Statistics 25.0 (IBM, Chicago, EEUU). Las variables categóricas se describen como valor absoluto (n) y porcentajes (%); la comparación de estas variables entre los grupos de pacientes leves y graves se realizó mediante la prueba de Chi-cuadrado de Pearson. Las variables continuas se expresan utilizando la mediana y el intervalo intercuartílico (IQR). Estas variables fueron comparadas en ambos grupos utilizando la prueba de la t de Student si la distribución era normal o la prueba *U* de Mann–Whitney si la distribución no era normal; la normalidad de las variables se estableció mediante la prueba de Kolmogorov–Smirnov.

Se realizaron análisis univariantes y multivariantes de factores pronósticos utilizando la regresión logística. En el análisis multivariante se empleó el método «pasos sucesivos hacia atrás (condicional)», incluyendo en el paso inicial todas las variables que mostraron una asociación univariante con un valor p<0,05. Como criterio de salida para la sucesiva exclusión de variables del modelo multivariante se estableció un valor p>0,10. El riesgo de progresión de la enfermedad atribuido a cada variable se expresa mediante la odds ratio (OR), junto al intervalo de confianza (IC) al 95%.

Para establecer el punto de corte de cada variable utilizado en la regresión logística se analizaron diferentes valores cercanos al punto de corte con mayor índice de Youden. Para ello, se dicotomizaron las variables estudiadas utilizando puntos de corte con números redondos. La selección de los puntos de corte utilizados en la regresión logística se realizó seleccionando aquellos que daban lugar a un modelo estadístico con mayor verosimilitud.

El área bajo la curva (AUC), la sensibilidad y la especificidad de los parámetros incluidos en la regresión logística se analizó mediante la curva de la característica operativa del receptor (ROC). En todos los análisis estadísticos realizados, el nivel de significación estadística se estableció en un valor p<0,05.

## Resultados

La población de este estudio incluyó a 197 pacientes con edades comprendidas entre 10 y 98 años (mediana=72), siendo 97 hombres y 100 mujeres. La distribución por sexos en los dos grupos estudiados fue similar, mientras que para la edad hubo diferencias estadísticamente significativas, siendo la mediana en el grupo de pacientes leves de 67 años y en el grupo de pacientes graves de 78 años.

El 64,47% (127/197) se consideraron pacientes leves y el 35,53% (70/197) pacientes graves, de los cuales 49 fueron exitus y 21 requirieron ingreso en la UCI con intubación endotraqueal. De los pacientes que estuvieron en UCI, 12 de ellos recibieron el alta hospitalaria mientras que 9 fallecieron. Así, de manera general, el 29,44% (58/197) de los pacientes fueron exitus. La causa más frecuente de exitus fue parada cardiorrespiratoria o síndrome de distrés respiratorio agudo (SDRA).

La hipertensión arterial fue la comorbilidad más común (58,4%), seguida de las alteraciones en los niveles de lípidos en sangre (35,0%), enfermedades cardiovasculares (28,9%) y diabetes mellitus (27,4%). Hay diferencias estadísticamente significativas entre los dos grupos en la insuficiencia renal crónica, cáncer, enfermedad vascular y enfermedad hepática, siendo los porcentajes de estas comorbilidades superiores en el grupo de pacientes graves. La Tabla 1 muestra los datos demográficos y antecedentes personales de los pacientes.

**Tabla 1:** Datos demográficos y características clínicas de los pacientes.

	Total n=197	Leves n=127	Graves n=70	p
Edad, años, mediana (IQR)	72 (59–84)	67 (57–80)	78 (68–86)	0,001
Género (hombre/mujer)	97/100	58/69	39/31	0,177
Comorbilidades, n (%)				
Hipertensión arterial	115 (58,4)	70 (55,1)	45 (64,3)	0,272
Dislipemia	69 (35,0)	39 (30,7)	30 (42,9)	0,087
Enfermedad cardiovascular	57 (28,9)	32 (25,2)	25 (35,7)	0,237
Diabetes mellitus	54 (27,4)	31 (24,4)	23 (32,9)	0,203
Insuficiencia renal crónica	27 (13,7)	12 (9,4)	15 (21,4)	0,019
Cáncer	22 (11,2)	10 (7,9)	12 (17,1)	0,048
Enfermedad vascular	19 (9,6)	5 (3,9)	14 (20,0)	0,001
Enfermedad pulmonar	18 (9,2)	11 (8,7)	7 (10,0)	0,755
Patología tiroidea	18 (9,2)	9 (7,1)	9 (12,9)	0,179
Enfermedad hepática	18 (9,2)	7 (5,5)	11 (15,7)	0,017
Anemia	13 (6,6)	8 (6,3)	5 (7,1)	0,819

La duración promedio de la hospitalización fue de 8 días, aumentando hasta los 27 días en los pacientes que ingresaron en UCI; la mediana de la duración de la estancia en UCI fue de 20 días. El 83,8% de los pacientes desarrolló neumonía adquirida en la comunidad (NAC) bilateral a causa de la infección por SARS-CoV-2. El CURB-65 es un índice del grado de severidad utilizado en la NAC que tiene en cuenta la edad del paciente, la presencia de confusión, la frecuencia respiratoria, la concentración de nitrógeno ureico y las tensiones arteriales sistólica y diastólica. La mayoría de los pacientes con neumonía tenía un CURB-65 de 2 puntos, a pesar de que este porcentaje era mayor en los pacientes graves (52,5%) que en los pacientes leves (34,9%) (Tabla 2).

**Tabla 2:** Desarrollo de neumonía y tratamiento farmacológico empleado.

	Total n=197	Leves n=127	Graves n=70
Neumonía, n (%)	165 (83,8)	106 (83,5)	59 (84,3)
CURB-65 = 0	31 (18,8)	26 (24,6)	5 (8,5)
CURB-65 = 1	44 (26,7)	34 (32,0)	10 (17,0)
CURB-65 = 2	68 (41,2)	37 (34,9)	31 (52,5)
CURB-65 = 3	22 (13,3)	9 (8,5)	13 (22,0)
Tratamiento, n (%)			
Hidroxicloroquina	161 (81,7)	110 (86,6)	51 (72,9)
Lopinavir/Ritonavir	94 (47,7)	62 (48,8)	32 (45,7)
Corticoides	58 (29,4)	24 (18,9)	34 (48,6)
Azitromicina	27 (13,7)	7 (5,5)	20 (28,6)
Tocilizumab	12 (6,1)	0 (0,0)	12 (17,1)
Interferón β	10 (5,1)	5 (3,9)	5 (7,1)
Ciclosporina	4 (2,0)	3 (2,4)	1 (1,4)
Anakinra	3 (1,5)	1 (0,8)	2 (2,9)

Durante el ingreso hospitalario de los pacientes incluidos en este estudio no existía evidencia científica procedente de ensayos clínicos controlados para recomendar algún tratamiento específico para SARS-CoV-2, por lo que los medicamentos administrados estaban en fase de investigación y su utilización seguía criterios incluidos en el protocolo de manejo clínico del hospital. El medicamento más utilizado en los pacientes con COVID-19 durante el ingreso hospitalario fue hidroxicloroquina (81,7%), seguido de la asociación de lopinavir y ritonavir (47,7%) y del grupo de corticoides (29,4%). Tanto azitromicina como tocilizumab se reservaron para los casos de mal pronóstico, en los que el tratamiento de base no presentaba beneficios en la mejora clínica de los pacientes (Tabla 2).

La Tabla 3 muestra los resultados de las pruebas de laboratorio en los dos grupos estudiados. Existen diferencias estadísticamente significativas entre pacientes leves y graves en parámetros hematológicos (leucocitos, neutrófilos, hemoglobina, VSG), hemostásicos (TP, TTPA, dímero D) y bioquímicos (creatinina, bilirrubina total, GGT, LDH, PCR, PCT, ferritina). No se encontraron diferencias estadísticamente significativas entre los dos grupos en los siguientes parámetros analíticos analizados: linfocitos, plaquetas y transaminasas (ALT y AST).

**Tabla 3:** Resultados de laboratorio en el momento del ingreso hospitalario.

	Total n=197	Leves n=127	Graves n=70	p
Parámetros analíticos, mediana, IQR				
Leucocitos, × 10^9^/L	6,76 (4,78–9,10)	6,15 (4,30–7,84)	8,74 (6,14–11,93)	<0,001
Linfocitos, × 10^9^/L	0,96 (0,75–1,34)	1,01 (0,77–1,31)	0,91 (0,63–1,44)	0,320
Neutrófilos, × 10^9^/L	5,35 (3,15–7,48)	4,49 (2,79–6,25)	7,23 (4,34–10,67)	<0,001
Hemoglobina, g/L	131 (119–144)	133 (121–146)	126 (115–137)	0,004
Plaquetas, × 10^9^/L	203 (158–271)	201 (157–269)	209 (160–275)	0,496
VSG, mm/h	47 (16–70)	36 (15–64)	56 (19–84)	0,024
TP, s	12,0 (11,4–13,0)	11,9 (11,4–12,6)	12,6 (11,8–13,7)	0,002
TTPA, s	32,1 (28,8–37,0)	31,4 (28,5–36,0)	33,4 (29,8–38,2)	0,047
Dímero D, mg/L	0,91 (0,54–1,92)	0,80 (0,47–1,38)	1,54 (0,71–3,42)	<0,001
Creatinina, µmol/L	83,1 (63,6–115,8)	76,9 (63,6–104,3)	107,9 (72,5–136,1)	0,001
Bilirrubina total, µmol/L	6,8 (5,1–10,3)	6,8 (5,1–10,3)	7,7 (5,1–12,5)	0,009
ALT, U/L	23,0 (14,0–34,4)	23,0 (14,1–34,0)	23,0 (13,6–37,0)	0,968
AST, U/L	33 (23–50)	31 (23–46)	36 (23–54)	0,331
GGT, U/L	39 (23–86)	31 (21–67)	54 (29–125)	0,002
LDH, U/L	294 (238–411)	288 (234–365)	322 (242–538)	0,023
PCR, mg/L	69,3 (24,4–140,8)	51,3 (17,9–120,5)	117,6 (50,2–247,8)	<0,001
PCT, ng/mL	0,12 (0,08–0,29)	0,09 (0,06–0,14)	0,31 (0,16–0,73)	<0,001
Ferritina, µg/L	507 (244–1052)	462 (224–781)	677 (276–1335)	0,012

VSG, velocidad de sedimentación globular; TP, tiempo de protrombina; TTPA, tiempo de tromboplastina parcial activada; ALT, alanina aminotransferasa; AST, aspartato aminotransferasa; GGT, γ-glutamiltransferasa; LDH, lactato deshidrogenasa; PCR, proteína C reactiva; PCT, procalcitonina.

En el análisis multivariante, los parámetros en el momento del ingreso hospitalario con mayor asociación a la gravedad de la infección por SARS-CoV-2 fueron una edad igual o superior a 70 años (OR=2,277; IC 95% 1,022–5,076), leucocitos≥9,5 × 10^9^/L (OR=6,577; IC 95% 2,605–16,608), PCR≥90 mg/L (OR=2,779; IC 95% 1,299–5,949), GGT≥30 U/L (OR=2,440; IC 95% 1,062–5,604) y PCT≥0,5 ng/mL (OR=11,590; IC 95% 3,439–39,054). En el modelo de regresión logística, la PCT presentó el mayor coeficiente, seguido de la concentración de leucocitos.

En el análisis univariante también se observa que la PCT es el parámetro con mayor asociación con la gravedad de la infección por SARS-CoV-2 (OR=21,750; IC 95% 7,215–65,571). Los resultados completos de la regresión logística se muestran en la Tabla 4.

**Tabla 4:** Regresión logística de los factores pronósticos de gravedad.

	Análisis univariante			Análisis multivariante		
	β	OR (IC 95%)	p	β	OR (IC 95%)	p
Edad, años						
<70		1 (ref)			1 (ref)	
≥70	1,129	3,094 (1,645–5,819)	<0,001	0,823	2,277 (1,022–5,076)	0,044
Leucocitos, × 10^9^/L						
<9,5		1 (ref)			1 (ref)	
≥9,5	2,241	9,405 (4,328–20,441)	<0,001	1,884	6,577 (2,605–16,608)	<0,001
PCR, mg/L						
<90		1 (ref)			1 (ref)	
≥90	1,528	4,611 (2,467–8,616)	<0,001	1,022	2,779 (1,299–5,949)	0,008
GGT, U/L						
<30		1 (ref)			1 (ref)	
≥30	1,027	2,792 (1,461–5,337)	0,002	0,892	2,440 (1,062–5,604)	0,036
PCT, ng/mL						
<0,5		1 (ref)			1 (ref)	
≥0,5	3,080	21,750 (7,215–65,571)	<0,001	2,450	11,590 (3,439–39,054)	<0,001

OR, odds ratio; IC, intervalo de confianza; PCR, proteína C reactiva; GGT, γ-glutamiltransferasa; PCT, procalcitonina.

**Figura 1:**
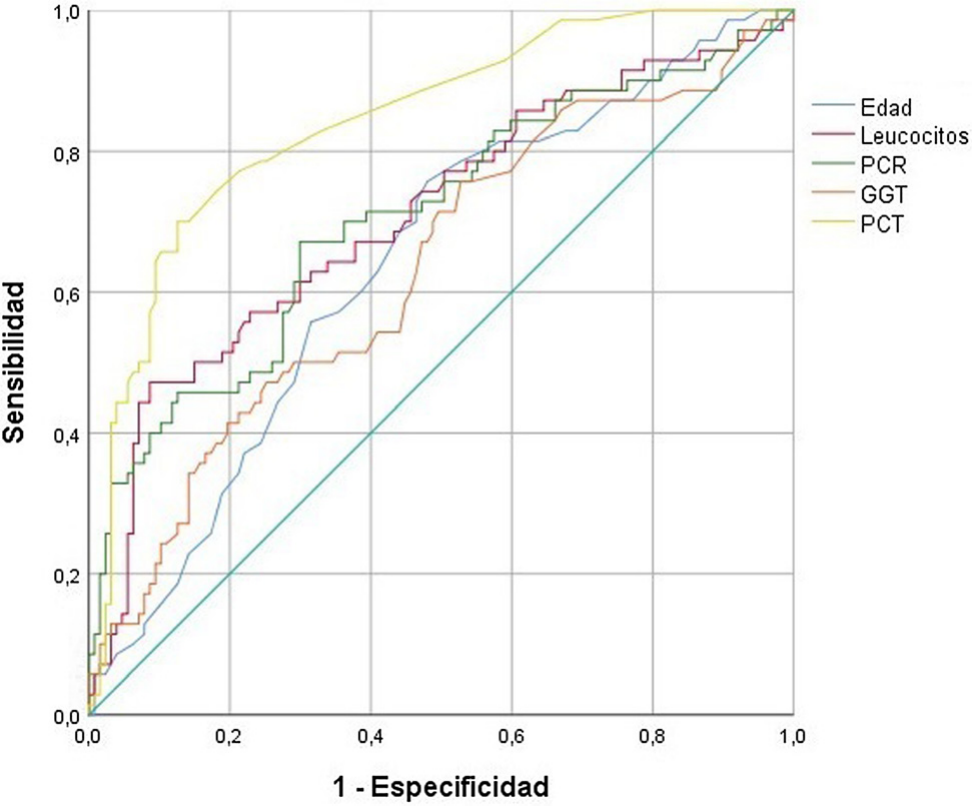
Curva ROC de los factores pronósticos de gravedad. PCR, proteína C reactiva; GGT, γ-glutamiltransferasa; PCT, procalcitonina.

La curva ROC de los factores pronósticos de gravedad incluidos en el análisis multivariante se muestra en la Figura 1, mientras que el AUC se encuentra en la Tabla 5.

**Tabla 5:** Área bajo la curva de los factores pronósticos de gravedad.

	AUC	IC 95%	p
Edad	0,642	0,562–0,721	0,001
Leucocitos	0,708	0,630–0,787	<0,001
PCR	0,708	0,629–0,786	<0,001
GGT	0,632	0,550–0,714	0,002
PCT	0,844	0,787–0,901	<0,001

AUC, área bajo la curva; IC, intervalo de confianza; PCR, proteína C reactiva; GGT, γ-glutamiltransferasa; PCT, procalcitonina.

Los puntos de corte con mayor índice de Youden obtenidos mediante la curva ROC fueron: edad≥67 años (sensibilidad 75,7% y especificidad 52,0%), leucocitos≥9,44 × 10^9^/L (sensibilidad 47,1% y especificidad 91,3%), PCR≥90,5 mg/L (sensibilidad 67,1% y especificidad 70,1%), GGT≥28 U/L (sensibilidad 75,7% y especificidad 47,2%) y PCT≥0,19 ng/mL (sensibilidad 70,0% y especificidad 87,4%).

## Discusión

Este trabajo ha identificado factores pronósticos de progresión de la infección por SARS-CoV-2 en pacientes hospitalizados. En concreto, una edad avanzada (≥70 años), niveles aumentados de leucocitos (≥9,5 × 10^9^/L) y concentraciones elevadas de PCR (≥90 mg/L), GGT (≥30 U/L) y PCT (≥0,5 ng/mL) en el momento del ingreso hospitalario se relacionan con una mayor probabilidad de presentar una forma grave de COVID-19.

Estudios anteriormente publicados han comunicado una media o mediana de edad variable en los pacientes con infección por SARS-CoV-2 (47–62 años) y la hipertensión como la comorbilidad más frecuente [9], [10]. En este estudio, la mediana de edad de los pacientes ingresados con infección por SARS-CoV-2 fue de 72 años y más de la mitad de los pacientes (58,4%) eran hipertensos (Tabla 1). En concreto, distintos estudios han comunicado que la edad avanzada es un importante predictor de mortalidad en SARS-CoV y MERS-CoV. Este trabajo confirma que el incremento de la edad se asocia con un pronóstico grave de la infección por SARS-CoV-2 [9], [10], [11].

Los resultados de este estudio indican que el recuento de glóbulos blancos y la concentración de PCT fueron superiores en pacientes con pronóstico grave. Esto coincide con otros estudios que indican que en pacientes que acaban falleciendo se debilita el sistema inmune innato, provocado por un cuadro infeccioso bacteriano o viral secundario a la infección por SARS-CoV-2 que causa un incremento de la serie blanca. A pesar de que la síntesis de PCT está inhibida por el interferón gamma que se produce en infecciones virales, el incremento de este biomarcador en el momento del ingreso hospitalario aumenta el riesgo de desarrollar una futura infección bacteriana [12], [13].

La PCR es un reactante de fase aguda que es empleado como marcador inflamatorio en la práctica clínica actual. Su síntesis es predominantemente hepática, bajo control transcripcional por la interleucina-6 que es originada en el foco inflamatorio. Distintos estudios han comunicado niveles elevados de PCR como un parámetro pronóstico de gravedad en infección por SARS-CoV-2 al estar relacionado con la progresión de los infiltrados pulmonares [9], [10], [11].

Han sido publicados diversos estudios que manifiestan la alteración de la función hepática en pacientes con infección por SARS-CoV-2 severa [14], [15], [16]. Este hallazgo sugiere que los hepatocitos y colangiocitos constituyen dianas potenciales de la infección por SARS-CoV-2 debido a que expresan el receptor ECA2, sobre el que actúa directamente el virus. Además, se ha demostrado una mayor susceptibilidad a esta infección en pacientes con disfunción hepática preexistente debido a un incremento en la expresión de ECA2, lo que obliga a una monitorización más exhaustiva de la función hepática [14], [15]. El aumento de la concentración de indicadores bioquímicos, entre los que se encuentra la GGT, en el momento del ingreso hospitalario es mayor en pacientes graves, coincidiendo los resultados de este trabajo con diversos estudios publicados [15], [16].

A pesar de que el CURB-65 se utiliza para predecir la mortalidad en pacientes con NAC, la mayoría de los pacientes ingresados con neumonía presentaban entre 0 y 2 puntos en esta escala (riesgo bajo o intermedio), incluidos aquellos que necesitaron ingreso en UCI o fallecieron. Sólo el 22,0% de los pacientes graves tenía un CURB-65 de 3 puntos (riesgo grave). Esto sugiere que la escala CURB-65 puede no ser adecuada para predecir la gravedad en pacientes con COVID-19 [17].

Los factores pronósticos de gravedad de COVID-19 descritos anteriormente presentan la ventaja de ser empleados frecuentemente en la práctica clínica y estar disponibles en todos los hospitales, permitiendo así disponer de los resultados analíticos de forma fácil y rápida en analizadores automatizados.

Este estudio presenta algunas limitaciones como son que el incremento de los glóbulos blancos y concentraciones elevadas de PCR, GGT y PCT pueden encontrarse en otras enfermedades inflamatorias e infecciosas. Además, el tamaño relativamente pequeño de la muestra puede dar lugar a resultados sesgados. Debido a esto, sería necesario un estudio multicéntrico a gran escala para confirmar los resultados obtenidos.

En conclusión, la edad avanzada, niveles elevados de leucocitos y concentraciones elevadas de PCR, GGT y PCT en el momento del ingreso hospitalario han sido identificados como predictores de gravedad en pacientes con infección por SARS-CoV-2 y pueden ser empleados para predecir el curso de la COVID-19 en las etapas iniciales, permitiendo adaptar la asistencia sanitaria a la estimación pronóstica de la enfermedad.
